# Chemical characterization by GC-MS and *in vitro* activity against *Candida albicans* of volatile fractions prepared from *Artemisia dracunculus*, *Artemisia abrotanum*, *Artemisia absinthium* and *Artemisia vulgaris*

**DOI:** 10.1186/1752-153X-8-6

**Published:** 2014-01-29

**Authors:** Diana Obistioiu, Romeo T Cristina, Ivo Schmerold, Remigius Chizzola, Klaus Stolze, Ileana Nichita, Viorica Chiurciu

**Affiliations:** 1 Banat’s University of Agricultural Sciences and Veterinary Medicine from Timisoara, Faculty of Veterinary Medicine, Calea Aradului no 119, Timisoara 300645, Romania; 2 Department for Biomedical Sciences, Institute of Pharmacology and Toxicology, University of Veterinary Medicine Vienna, Vienna A-1210, Austria; 3 Institute for Applied Botany and Pharmacognosy, University of Veterinary Medicine Vienna, Vienna A - 1210, Austria; 4 Romvac, Voluntari, Bucuresti 061182, Romania

**Keywords:** *Artemisia*, Essential oils, GC-MS, Antifungal activity, *Candida albicans*

## Abstract

**Background:**

A large number of essential oils is reported to have significant activity against *Candida albicans*. But the different chemical composition influences the degree of their activity. The intention of this study was to investigate the chemical composition and the activity against *Candida albicans* of volatile oils obtained from *Artemisia dracunculus*, *A. abrotanum*, *A. absinthium* and *A. vulgaris* (*Asteraceae*). The aim of the study was to identify new chemical compounds that have effect against *C. albicans*.

The essential oils were obtained by hydrodistillation or extraction with dichloromethane (a new procedure we developed trying to obtain better, more separated compounds) from air dried above ground plant material and analyzed by GC-MS. Additionally commercial essential oils from the same species were tested. The *Candida albicans* inhibition studies were carried out by the paper disc diffusion method.

**Results:**

The essential oils shared common components but presented differences in composition and showed variable antifungal activity. Davanone and derivatives thereof, compounds with silphiperfolane skeleton, estragole, davanone oil, β-thujone, sabinyl acetate, herniarin, cis-chrysanthenyl acetate, 1,8-cineol, and terpineol were the main components of *Artemisia* volatiles.

**Conclusions:**

Among the volatile fractions tested those from *A. abrotanum* containing davanone or silphiperfolane derivatives showed the highest antifungal activity. The *in vitro* tests revealed that the *Artemisia* oils are promising candidates for further research to develop novel anti-candida drugs.

## Background

The genus *Artemisia* belongs to one of the largest and most widely distributed genera of the family *Asteraceae* (*Compositae*). It is a diverse and economically important genus and it has more than 500 species. Most plants within this genus have a great importance as medication, foodstuff, ornamentals or soil stabilizers, some are allergenic or toxic, and some are weeds growing in the fields [1-4].

Antimycotic activity of *A. absinthium* against *Candida albicans* was reported in several in vitro studies [5,6] however, there are also investigations that found no such activity [7-10].

The conditions that may separate these studies are the plant preparations put under investigation. The highest percentage of successful antifungal activity of *Artemisia* plants was obtained with essential oils; raw extracts generally showed less activity [5-10].

More information on the potential antifungal activity of *Artemisia* plants could stimulate research leading to their possible future use in the clinical treatment of candidiasis as natural antimycotic agents.

## Results and discussion

In the present study, the chemical composition and the antifungal in vitro potency of seven oil preparations obtained from air dried plants and commercial essential oil samples of 4 different *Artemisia* species were put under investigation. Their chemical composition was evaluated by GC-MS. The antifungal activity was determined by the disk diffusion method test. The tests were done using the disc diffusion method following CLSI standard rules for antimicrobial susceptibility testing of impregnated disks [11].

### Chemical composition

The volatile fractions studied showed a great chemical diversity. All four *Artemisia* species had distinct volatile patterns and there were also differences in the composition between the two different extracts, the distilled oil and the CH_2_Cl_2_ extract, of the same species, as presented in Table 1.

**Table 1:** **GS**-**MS chemical composition of the volatiles from the investigated *Artemisia *** **species**

**No**	**RI**	**Compound**	***A. abrotanum *** **plant % (mg/ml)**	***A. abrotanum *** **CH**_**2**_**Cl**_**2**_**% (mg/ml)**	***A. dracunculus *** **plant % (mg/ml)**	***A. dracunculus *** **CH**_**2**_**Cl**_**2**_**% (mg/ml)**	***A. absinthium *** **CH**_**2**_**Cl**_**2**_% (**mg**/**ml**)	***A. vulgaris *** **plant % (mg/ml)**	***A. vulgaris *% (mg/ml)**
**1.**	969	4-Methyl-pent-2-enolid	15.7 (1.7869)	1.7 (0.1379)	-	1.8 (0.046)	-	-	-
**2.**	991	1-Octen-3ol	-	-	-	-	-	-	4.2 (0.253)
**3.**	1037	1,8-Cineol	-	-	-	-	-	-	18 (1.077)
**4.**	1043	*Z*-β-Ocimene	-	-	1.3 (0.103)	-	-	-	-
**5.**	1044	Lavender lactone	2.6 (0.3017)	-	-	-	-	-	-
**6.**	1053	*E*-β-Ocimene	-	-	1.6 (0.124)	-	-	-	-
**7.**	1069	*cis*-Arbusculone	0.7 (0.0885)	-	-	-	-	-	-
**8.**	1077	*trans*-Arbusculone	0.6 (0.0738)	-	-	-	-	-	-
**9.**	1100	*trans*-Sabinene hydrate	-	-	-	-	-	3.5 (0.079)	-
**10.**	1114	Phenylethyl alcohol	-	-	-	-	-	1.8 (0.042)	2.1 (0.129)
**11.**	1122	β-Thujone	-	-	-	-	14.7 (0.380)	-	-
**12.**	1129	1-Terpineol	1.6 (0.1817)	-	-	-	-	-	-
**13.**	1132	*allo* Ocimene	-	-	0.3 (0.028)	-	-	-	-
**14.**	1141	*iso*-3-Thujanol	-	-	-	-	2.2 (0.059)	-	-
**15.**	1147	*cis*-β-Terpineol	1.2 (0.1402)	0.8 (0.0700)	-	-	-	-	-
**16.**	1152	Camphor	-	-	-	-	-	-	5.6 (0.337)
**17.**	1173	Borneol	-	-	-	-	-	5.4 (0.122)	13.2 (0.792)
**18.**	1183	Terpinen-4-ol	-	-	-	-	-	18.2 (0.407)	13.5 (0.801)
**19.**	1189	*p*-Cymen-8-ol	-	-	-	-	-	-	1.9 (0.113)
**20.**	1195	α-Terpineol	-	-	-	-	-	10 (0.223)	6.6 (0.394)
**21.**	1202	Estragole	0.9 (0.1123)	0.8 (0.0702)	84.1 (6.406)	-	5 (0.126)	-	
**22.**	1211	*trans*-Piperitol	1.2 (0.1454)	-	-	-	-	-	-
**23.**	1221	*trans*-Carveole	-	-	-	-	-	1.9 (0.043)	-
**24.**	1226	Nerol	-	-	-	-	11.5 (0.297)	-	-
**25.**	1227	Nordavanone,	3 (0.3414)	5.4 (0.4275)	-	8.3 (0.206)	-	-	-
**26.**	1252	Geraniol	-	-	-	-	2.6 (0.069)	-	-
**27.**	1253	Chavicol	-		0.4 (0.031)	1 (0.023)	-	-	-
**28.**	1257	Piperitone	0.5 (0.0616)	-	-	-	-	-	-
**29.**	1260	*cis*-Chrysanthenylacetate	-	-	-	-	2.5 (0.065)	-	15 (0.897)
**30.**	1292	Cuminol	-	-	-	-		7.2 (0.163)	1.7 (0.105)
**31.**	1299	UnknownABS					29.1 (0.750)		
**32.**	1331	1.4-*p*-Menthadien-7-ol	-	-	-	-		3.6 (0.082)	1.7 (0.105)
**33.**	1354	Eugenol	-		1.4 (0.107)	1.4 (0.036)	0.9 (0.023)	3.8 (0.086)	3 (0.175)
**34.**	1367	UnknownABR1		5.2 (0.4115)	-	-	-	-	-
**35.**	1386	*E*-Methylcinnamate	-	-	0.9 (0.068)	-	-	-	-
**36.**	1399	Methyleugenol	-		2.2 (0.173)	1.4 (0.035)	1.5 (0.040)	-	-
**37.**	1401	UnknownABR2	1.3 (0.1540)						
**38.**	1422	UnknownABR3		20.7 (1.6363)		4.3 (0.106)			
**39.**	1466	γ-Decalactone	-	-	0.2 (0.015)	-	-	-	-
**40.**	1483	Germacrene D	-	-	0.2 (0.015)	-	-	0.5 (0.011)	1.6 (0.091)
**41.**	1484	Davanone ether	0.9 (0.1016)	-	-	-	-	-	
**42.**	1486	Farnesene	-	-	-	-	-	-	1(0.062)
**43.**	1491	β-Selinene	-	-	-	-	-	-	1.2 (0.076)
**44.**	1500	Bicyclogermacrene	-	-	0.1 (0.012)	-	-	-	-
**45.**	1504	Davana ether*	3.2 (0.3591)	-	-	-	-	-	-
**46.**	1518	Artedouglasia oxide C	1.7 (0.1932)	1.1 (0.0869)		1.7 (0.042)			
**47.**	1520	δ-Cadinene	-	-	-	-	-	-	1.5 (0.093)
**48.**	1522	Davana ether*	1.4 (0.1581)	-	-	-	-	-	-
**49.**	1531	Artedouglasia oxide A	2.2 (0.2575)	1.8 (0.1486)	-	3.4 (0.084)	-	-	-
**50.**	1554	Artedouglasia oxide D	1 (0.1201)	0.6 (0.0534)	-	1 (0.023)	-	-	-
**51.**	1476	Artedouglasia oxide B	1.3 (0.1469)	1 (0.0858)	-	1.6 (0.040)	-	-	-
**52.**	1580	*cis*-Davanone	5.2 (0.5989)	7.4 (0.5896)	0.6 (0.048)	9.1 (0.225)	-	-	-
**53.**	1589	Spathulenol	-	-	-	-	6.5 (0.168)	12.1 (0.271)	7.1 (0.425)
**54.**	1585	Caryophyllene oxide	-	-	-	-	2.8 (0.072)	8.5 (0.191)	2.7 (0.163)
**55.**	1598	UnknownABR4	-	2 (0.1582)	-	-	-	-	-
**56.**	1599	UnknownABR5	1.3 (0.1570)	-	-	-	-	-	-
**57.**	1641	UnknownABR6	2.7 (0.3121)	-	-	-	-	-	-
**58.**	1655	Bisabololoxide B	-	-	-	-	3.3 (0.086)	-	-
**59.**	1656	epi-α Muurolol	-	-	-	-	-	11.8 (0.265)	-
**60.**	1684	α-Bisabolol	-	-	-		11 (0.285)	-	-
**61.**	1685	UnknownABR7	12.4 (1.4113)	17.2 (1.3599)	0.5 (0.041)	20.5 (0.506)	-	-	-
**62.**	1688	UnknownABR8	4 (0.4598)	-	-	-	-	-	-
**63.**	1711	UnknownABR9	8.7 (0.9892)	-	-	3.4 (0.085)	-	-	-
**64.**	1719	Herniarin,	-	-	4.3 (0.329)	10 (0.246)	5.8 (0.150)	-	-
**65.**	1754	Davanone Derivative	22.5 (2.5593)	33.3 (2.6535)	-	18.2 (0.450)	-	-	-
**66.**	1936	UnknownDRA	-	-	0.3 (0.029)	7 (0.174)	-	-	-
**67.**	1963	Hexadecanoic acid	-	-	-	-	-	11.2 (0.249)	-
**68.**	2107	Phytol-isomer	1.2 (0.1451)	-	1.1 (0.087)	(0.138)	-	-	-

* exact isomer not determined, RI retention index.

Mass spectra of the unidentified compounds.

RI 1367 UnknownABR1: 41 (50), 43 (72), 44 (13), 45 (14), 53 (15), 55 (100), 56 (13), 57 (30), 67 (32), 68 (13), 69 (37), 71 (41), 74 (11), 81 (19), 83 (14), 84 (11), 85 (11), 93 (37), 95 (25), 111 (34), 123 (10), 169 (46).

RI 1299 UnknownABS: 41 (39), 43 (68), 44 (8), 53 (16), 55 (22), 57 (14), 59 (100), 67 (49), 68 (19), 69 (9), 71 (34), 77 (11), 79 (32), 81 (45), 82 (55), 83 (12), 89 (11), 93 (7), 94 (7), 109 (10), 152 (11).

RI 11401 UnknownABR2: 55 (100), 41 (70), 43 (100), 44 (25), 53 (16), 56 (14), 57 (55), 59 (26), 67 (31), 69 (44), 71 (34), 77 (12), 81 (29), 83 (26), 85 (19), 91 (18), 93 (96), 95 (15), 97 (20), 111 (68), 125 (15), 126 (26).

RI 1422 UnknownABR3: 41 (32), 43 (62), 53 (13), 55 (83), 57 (10), 67 (24), 68 (14), 69 (28), 71 (100), 72 (14), 77 (10), 81 (24), 83 (16), 91 (14), 93 (74), 95 (9), 100 (19), 110 (11), 111 (94), 125 (18), 195 (11).

RI 1598 UnknownABR4: 41 (9), 42 (3), 43 (10), 44 (3), 53 (4), 55 (13), 57 (6), 67 (5), 68 (3), 69 (5), 71 (4), 79 (2), 81 (4), 83 (5), 85 (100), 86 (5), 93 (9), 107 (2), 111 (3), 114 (12), 156 (4).

RI 1599 UnknownABR5: 41 (38), 43 (100), 44 (14), 55 (30), 57 (25), 69 (22), 71 (17), 77 (14), 79 (20), 81 (32), 83 (22), 85 (20), 91 (15), 93 (18), 95 (17), 109 (45), 149 (14), 161 (15), 207 (46), 219 (21), 234 (15).

RI 1641 UnknownABR6: 41 (52), 43 (68),44 (13), 53 (18), 55 (100), 56 (13), 57 (29), 67 (27), 68 (18), 69 (37), 71 (23), 79 (12), 81 (27), 83 (32), 84 (17), 85 (12), 91 (12), 93 (72), 95 (24), 109 (13), 111 (73).

RI 1685 UnknownABR7: 41 (38), 43 (40), 53 (9), 55 (53), 57 (10), 67 (15), 68 (10), 69 (43), 71 (18), 77 (6), 81 (17), 83 (20), 91 (9), 93 (64), 94 (7), 95 (8), 97 (9), 111 (100), 112 (12), 125 (19), 140 (13).

RI 1688 UnknownABR8: 41 (62), 43 (100), 53 (15), 55 (82), 57 (23), 67 (31), 68 (28), 69 (43), 70 (16), 71 (77), 72 (19), 79 (12), 81 (31), 82 (15), 83 (20), 91 (13), 93 (93), 94 (14), 95 (24), 110 (15), 111 (76), 113 (13), 125 (20).

RI 1711 UnknownABR9: 43 (100), 93 (66), 55 (55), 111 (55), 113 (51), 85 (39), 41 (35), 69 (30), 67 (26), 95 (25), 81 (24), 125 (20), 57 (20), 71 (19), 97 (17), 96 (17), 109 (14), 83 (14), 68 (14), 138 (11), 53 (11), 236 (5).

RI 1754 Davanone-derivative: 41 (45). 43 (100), 53 (17), 55 (74), 57 (24), 67 (32), 68 (14), 69 (45), 71 (36), 79 (18), 81 (48), 82 (12), 83 (23), 91 (12), 93 (87), 95 (20), 96 (29), 97 (48), 109 (20), 111 (90), 112 (12), 113 (15), 125 (69), 236 (36).

RI 136 UnknownDRA: 41 (40), 43 (12), 53 (22), 55 (23), 57 (17), 66 (23), 67 (28), 69 (25), 77 (12), 79 (18), 81 (84), 82 (9), 83 (12), 95 (21), 96 (74), 98 (14), 110 (17), 113 (13), 151 (100), 152 (37), 22.

*A. abrotanum* was characterized by the high content of the sesquiterpene davanone and its derivatives which could not all be identified as well as by the artedouglasia oxides A-D. Monoterpenes like 1-terpineol or *trans*-piperitol and the phenylpropanoid estragole were present in small amounts only in this species. All main compounds found in the distilled oil were also present in the CH_2_Cl_2_ extract. The davanone derivatives were also the main compounds in the microdiostillates which also contained the artedouglasia oxides.

The main compound in the distilled oil of *A. dracunculus* was estragole accounting for approximately 80% of the oil. However, in the CH_2_Cl_2_ extract this compound could not be identified. Small amounts of the phenylpropanoids chavicol and methyleugenol could be found. These extracts contained also some davanone and its derivatives. Both, the distilled oil and the extract contained the coumarin derivative herniarin. In the microdistillate, the main compounds were estragole (79.5%) followed by *Z*-β-ocimene (8.8%) and *E*-β-ocimene (7.2%).

From *A. absinthium* only a CH_2_Cl_2_ extract was obtained. The main peak in the chromatogram could not be identified. Furthermore, the extract contained β-thujone and nerol as major compounds and estragole, spathulenol and α-bisabolol as further components. Herniarin was also present in this extract. The microdistillate was characterized by the presence of β-thujone (78.1%), sabinene (4.4%), and myrcene (4.4%).

The main volatiles in *A. vulgaris* were terpinen-4-ol, and borneol. One noticeable difference between the two volatile preparations was that 1,8-cineol, the main compound in the oil from the CH_2_Cl_2_ extract, was not found in the distilled oil.

The composition of the commercial *Artemisia* essential oils samples is displayed in Table 2. In contrast to the own preparations of *A. abrotanum* oils, the commercial sample was dominated by the sesquiterpene silphiperfolene derivatives and contained appreciable amounts of 1,8-cineol (21.7%, 168 mg/ml), borneol (8.2%, 64 mg/ml) and *p*-cymene (6.9%, 54 mg/ml). The *A. dracunculus* sample had estragol as main compound and the ocimene isomers similar to the microdistillation fingerprint of the own material from the botanical garden. The commercial *A. absinthium* sample had β-thujone (41%, 358 mg/g), sabinyl acetate (22%, 153 mg/g) and α-thujone (13%, 113 mg/ml) as main compounds. Finally, the purchased *A. vulgaris* essential oil was rich in α-thujone (65%, 531 mg/g), camphor (14%, 110 mg/g), and β-thujone (10%, 79 mg/g).

**Table 2:** **Composition of the commercial *Artemisia *** **essential oils** (**mg**/**ml**)

**No**	**RI**	**Compound**	***A. abrotanum *% (mg/ml)**	***A. dracunculus *% (mg/ml)**	***A. absinthium *% (mg/ml)**	***A. vulgaris *% (mg/ml)**
**1**	800	Octane	-	0.1 (0.8)	01. (0.9)	
**2**	927	Tricyclene	0.08 (0.6)			0.1 (0.8)
**3**	932	α-Thujene	-			0.03 (0.3)
**4**	939	α-Pinene	0.1 (1.1)	1.4 (11.9)	0.3 (2.9)	0.1 (1.0)
**5**	953	Camphene	2.7 (18.5)	0.05 (0.5)	0.1 (1.2)	2.2 (18.0)
**6**	978	Sabinene	0.1 (0.9)	0.07 (0.6)	2 (16.1)	1.8 (15.2)
**7**	982	β-Pinene	0.3 (2.3)	0.1 (1.5)	0.3 (2.6)	0.2 (1.6)
**8**	993	Myrcene		0.1 (0.9)	6.4 (6.5)	
**9**	1008	α-Phellandrene			0.2 (1.6)	
**10**	1021	α-Terpinene	0.9 (6.4)			
**11**	1030	*p*-Cymene	7.8 (53.5)		0.1 (1.4)	0.7 (6.3)
**12**	1034	Limonene		4 (33.8)	0.4 (3.4)	
**13**	1037	1,8-Cineol	24.5 (167.9)		1.5 (12.3)	0.6 (5.1)
**14**	1042	*Z*-β-Ocimene		8.3 (69.5)		
**15**	1053	*E*-β-Ocimene		7.8 (65.5)		
**16**	1064	γ-Terpinene	0.4 (3.1)		0.1 (1.0)	
**17**	1073	*cis*-Sabinene hydrate	0.3 (2.0)			0.02 (0.2)
**18**	1092	Terpinolene	0.1 (0.7)			
**19**	1093	Fenchone		0.3 (3.0)		
**20**	1102	Linalool			2 (16.4)	
**21**	1102	*trans*-Sabinene hydrate	0.3 (2.4)			0.07 (0.6)
**22**	1110	α-Thujone			13. (112.5)	65.5 (530.5)
**23**	1122	β-Thujone			44 (357.6)	9 (73.2)
**24**	1126	Dehydro sabinaketone				1.5 (12.8)
**25**	1132	*allo*-Ocimene		0.1 (1.0)		
**26**	1136	*Z*-Epoxyocimene			1.5 (12.9)	
**27**	1146	*trans*-Sabinol			3 (24.2)	
**28**	1146	*trans*-Pinocarveol				0.5 (4.1)
**29**	1146	*neo*-*allo*-Ocimene		0.07 (0.6)		
**30**	1152	Camphor	3.5 (24.2)		0.8 (7.2)	13.6 (110.4)
**31**	1165	Sabina ketone				0.3 (2.4)
**32**	1171	Isoborneol			0.8 (6.6)	
**33**	1173	Borneol	9.3 (63.8)			0.4 (3.9)
**34**	1183	Terpinen-4-ol	1.8 (12.7)		0.3 (2.9)	0.5 (4.4)
**35**	1195	α-Terpineol	0.2 (1.5)			
**36**	1201	Myrtenal	0.37 (2.6)			0.4 (3.2)
**37**	1202	Estragol		75 (626.1)		
**38**	1235	Isobornylformate	0.1 (1.3)			
**39**	1240	Fenchyl acetate		0.1 (0.8)		
**40**	1247	*trans*-Chrysanthenyl acetate	1 (7.2)			
**41**	1248	Cuminal				0.1 (1.0)
**42**	1257	Piperitone				0.2 (1.6)
**43**	1266	Verbenyl acetate				0.4 (3.4)
**44**	1291	Bornyl acetate	0.7 (5.2)			0.07 (0.6)
**45**	1291	Anethol		1.5 (12.7)		
**46**	1292	Lavandulyl acetate			0.4 (3.7)	
**47**	1296	Sabinyl acetate			23.7 (193.2)	
**48**	1334	Silphiperfol-5-ene	0.7 (5.3)			
**49**	1346	Silphiperfolen-Isomer	0.5 (3.9)			
**50**	1353	7 β-H-Silphiperfol-5-ene	0.6 (4.4)			
**51**	1367	Silphiperfol-4, 7(14)-diene	0.3 (2.6)			
**52**	1369	Silphiperfolen-Isomer	0.7 (5.0)			
**53**	1384	α-Copaene			0.1 (1.4)	0.07 (0.6)
**54**	1408	*Z*-Methyl-isoeugenol		0.2 (2.2)		
**55**	1425	Linalyl isobutyrate			0.2 (1.7)	
**56**	1429	β-Caryophyllene	0.7 (5.3)	0.05 (0.5)	1.4 (11.8)	
**57**	1464	α-Humulene	1.8 (1.3)		0.1 (1.1)	
**58**	1483	Germacrene D	3.4 (23.5)	0.05 (0.5)		
**59**	1497	Farnesene		0.04 (0.4)		
**60**	1500	Bicyclogermacrene		0.05 (0.5)	0.3 (2.4)	
**61**	1507	Farnesene				0.03 (0.3)
**62**	1512	Lavandulyl isovalerate			0.7 (5.9)	
**63**	1513	Unknown	5.7 (39.4)			
**64**	1531	β-Sesquiphellandrene		0.05 (0.5)		
**65**	1531	Unknown	1.7 (12.1)	0.1 (0.9)		
**66**	1548	Siphiperfol-5-en-3-one B	2.7 (19.1)			
**67**	1558	Silphiperfol-5-en-3-one B-isomer	5.1 (35.2)			
**68**	1583	Silphiperfol-5-en-3-one A	18.9 (129.5)			
**69**	1589	Spathulenol				
**70**	1595	Unknown	3 (20.1)			

### Test microorganisms

The anticandidal test with the commercial oils is presented in Table 3. All four oils showed anticandidal activity with *A. abrotanum* being the most efficient, followed by *A. absinthium*, *A. dracunculus* and *A. vulgaris*.

**Table 3:** **Inhibitory in vitro effects of the commercial *Artemisia *** **essential oils on *Candida albicans *** (**ATCC 10231**)

**Essential oil**	**Inhibition zone diameter (mm** **±** **SD)**
*A. dracunculus*	15.5 ± 2.1
*A. abrotanum*	20.0 ± 1.4
*A. absinthium*	17.0 ± 1.4
*A. vulgaris*	12.5 ± 0.7
Nystatin (positive control)	15.0 ± 0
Blank filter paper (negative control)	6.0 ± 0

The four investigated *Artemisia* species revealed distinct volatile patterns. A comparison with published data showed that even within one and the same species the oil composition may vary widely, representing different chemotypes. For instance, *A. abrotanum* from Cuba had trans-sabinyl acetate and α-terpineol as main oil compounds [12]. The same species collected in Serbia displayed silphiperfol-5-en-3-one A (14.6%), ascaridole (13.1%), 1,8-cineole (10.5%), α-bisabolol oxide A acetate (8.7%) as main oil components [13]. Similarly, an oil from Northwestern Italy had 1,8-cineole (34.7%), bisabolol oxide (18.4%) and ascaridole (16.0%) as main compounds [14]. The dominant components in the oil from the Crimea were 1,8-cineole and camphor [15]. A German *A. abrotanum* showed 1,8-cineol as main oil compound [16]. Plant cultivated in Poland were rich in piperitone (17.5%), davanone (16.8%), 1,8-cineole (12.5%) and silphiperfol-5-en-3-ol A (6.3%). These plants contained also the artedouglasia oxides as minor compounds [17].

The present oil of *A. dracunculus* and the oils reported in literature were dominated by phenylpropanoids. A Polish oil sample contained elemicin (48.8%), sabinene (18.9%), isoelemicin (13.3%) and eugenol (7.6%) [17], different Finnish French tarragon contained *cis*- and *trans*-ocimenes, estragole and herniarin [18], and Crimean samples sabinene and elemicine [19]. Canadian *A. dracunculus* essential oil was mainly composed of methyl eugenol (35.8%), eugenol (16.2%) and terpinolene (19.1%) [20].

According to the literature [21], the essential oil composition of *A. absinthium L*. was obtained and examined from different geographical parts of Europe the *Absithii* herba (plant) can be divided into at least 4 chemotypes: thujones rich oil (Greece, Spain, Ukraine, France, Italy), sabinene acetate rich oil (Armenia, Latvia, Belgium, Lithuania), epoxyocimenes rich oil (Russia), and a chemotype in which oil monoterpenes sabinene and myrcene were predominant (Estonia, Scotland, Moldova, Hungary). This is partly in accordance with a former study [22] where 19 samples of *A. absinthium* from Italy, France, Romania, and Siberia were divided into four chemotypes: sabinyl acetate rich oil, epoxyocimenes rich oil, chrysanthenyl acetate rich oil, and thujone rich oil. An epoxy-ocimene rich oil was also found in northwestern Italy [14]. An oil rich in t-sabinylacetate, thujone and myrcene is reported from Canada [20]. Furthermore a Turkish *A. absinthium* oil had chamazulene and nuciferol esters as major compounds [23].

Cited literature [5,15] described an *A. absinthium* oil rich in thujone and in the aerial parts of *A.vulgaris* an oil with high proportions of 1,8-cineole, sabinene, thujone, and caryophyllene oxide.

Essential oils from *A. vulgaris* rich in camphor, 1,8 cineole or β-thujone have been described [14,24-26]. These oils contain also chrysanthenyl acetate, borneol, methyleugenol, α-terpineol, t-verbenol, or β caryophyllene but owing to the great variability present it is difficult to point out distinct chemotypes. The *A. vulgaris* volatile fraction in the present study contained terpinen-4-ol, borneol, cis-chrysanthenyl acetate, and spathulenol as major compounds.

In this study, all volatile fractions isolated from all four *Artemisia* species showed some activity against *Candida albicans*. The highest activity was found for *A. abrotanum* where an inhibition zone larger than that produced by Nystatin could be observed. But it has to be considered that these fractions contain higher amounts of potentially active compounds than the fractions obtained from the other investigated species. Nevertheless, there are reports confirming the antimycotic activity of the main compounds identified by GC-MS in the present volatile fractions.

An extract from *A. abrotanum* grown in Sweden as well as the component davanone were effective against *C. albicans*[27]. Furthermore, davana oil from *Artemisia pallens* rich in davanone and the davana fraction derived from this oil were active against *Candida albicans*[28]. However in another study, davanone as single compound showed only very weak *in vitro* activity against *C. albicans*[29]. *Candida albicans* proved to be very susceptible against the essential oil from *Ocimum selloi* which contains more than 95% estragole (= methylchavicol) [30]. However, in another test series, estragole as single substance proved to be ineffective [31].

In the present study, 1,8-cineol, borneol, terpinen-4-ol, spathulenol, β-thujone, and α-bisabolol were major compounds in all of the volatile fractions of *A. absinthium* and *A. vulgaris* studied. The oils containing these compounds can be found in various plants and some of them were reported to inhibit the growth of *C. albicans*. An essential oil rich in α-pinene and α-bisabolol from *Laserpitium zernyi* showed a low activity against two strains of *C. albicans*[32]. The oil of *A. annua* containing 48% camphor as main compound was active against *C. albicans*[33]. A sage (*Salvia officinalis*) oil from Montenegro with the main compounds α-thujone (29.5%), camphor (22.5%) and 1,8-cineole (12.2%) had significant activity against *C. albicans*[34].

Oils from sage, myrtle, and laurel containing 1,8-cineol as main compound showed some activity against *C. albicans*[35], also an *Eucalyptus* oil with 85% of 1,8-cineole content [36]. However an Egyptian oil from *Eucalyptus occidentalis* that presumably contained 1,8 cineole had no influence on the growth of *C. albicans*[37].

Additionally, 7-methoxycoumarin (herniarin) isolated from twigs of *Treculia obovoidea* showed some activity against various *Candida* species [38]. Although reports on the antifungal activity of *Artemisia* species are inconsistent the results of this study confirm that *Artemisia* essential oils exert antimycotic effects and may represent good candidates to replace in the future allopathic treatments to which *Candida* has developed resistance. Under the conditions of this study, volatile fractions from *A. abrotanum* as well as the commercial oil from this plant had the strongest effect on *Candida albicans* although they differed in composition, having either davanone derivatives or silphiperfolene derivatives and 1,8-cineol as major compounds, respectively. The differences in activity and order in efficacy of the oils and volatile preparations most probably arises from differences in composition. So the own *A. vulgaris* preparations containing a mix of 1,8-cineol, terpinen-4-ol, borneol, camphor, and spathulenol were more active than the commercial oil with the thujones and camphor. The lower activity of the *A. absinthium* dichloromethane extract might be due to the lower overall concentration of active compounds in this preparation as compared to the commercial sample from the same species.

### Experimental research

#### Plant material

The *A. abrotanum*, *A. dracunculus*, and *A. absinthium* aerial parts were collected in the Botanical Garden of the University of Veterinary Medicine Vienna, Austria. *A. vulgaris* aerial parts were gathered in the surroundings of Timisoara (Bencecul de Sus), Romania. All plants were picked during the June-September period of 2010, while blooming. Voucher specimen of the collected plants were deposited in the Herbarium of the University of Vienna (WU-Generale, http://http:\\herbarium.univie.ac.at).

#### Commercial essential oils

Additionally, other commercial essential oils from *Artemisia absinthium* (USA), *A. dracunculus* (Iran), and *A. vulgaris* (Marokko) were obtained from Baccara Rose (Dagmar Köhler, 47665 Sonsbeck, Germany). *Artemisia abrotanum* came from Ayus GmbH, 77815 Bühl/Moos, Germany.

#### Test microorganisms

Isolates of *Candida albicans* (ATCC 10231) were obtained from the culture collection of the Department of Microbiology, Banat’s University of Agricultural Sciences and Veterinary Medicine Timisoara.

## Methods

The preparation of the essential oils was done using a modified standard procedure according to the European Pharmacopoeia [39]. The procedures were done at the same period of time. The plant material (15 g) was subjected to hydrodistillation using 150 ml distilled water for 4 hours at a constant rate. An average of 18.5 mg of essential oil was obtained.

In addition, an extraction with dichloromethane (CH_2_Cl_2_) followed by hydrodistillation was carried out. 100 g of fresh plant material (leaves or flowers) were extracted 5 times with 100 ml of CH_2_Cl_2_ in consecutive steps. The resulting extracts were combined and the solvent was removed in vacuo using a Rotavapor. The average yield was 2 g of crude extract; the pooled extract was hydrodistillated for 3 hours as mentioned above and stored at 4˚C until analysis. An average yield of 30 mg oil out of 2 g extract was obtained. For further analyses volatile oil from several preparations were combined.

Leaves of the plants collected from the botanical garden were also subjected to microdistillation using the automatic microdistillation unit MicroDistiller from Eppendorf (Hamburg, Germany) which is a gentle distillation method that allows investigating essential oil finger prints. The procedure was as follows: 0.2 to 0.3 g finely crushed dried plant material and 10 ml distilled water were filled into the sample vial. The collecting vial containing 1 ml water, 0.5 g NaCl and 300 μl *n*-hexane was connected with a capillary to the sample vial. The heating program applied to the sample vial was 15 min at 108°C followed by 45 min at 112°C. The collecting vial was kept at -2°C, where the volatiles were trapped in 0.3 ml *n*-hexane.

### Analysis of the essential oils

The analysis of the volatiles was performed using a Hewlett-Packard 6890 GC linked to a Hewlett- Packard 5973 mass-selective detector. For the analysis a Zebron ZB-5MS, capillary column (27 m × 250 μm i. d., 0.25 μm film thickness) was used. The carrier gas was helium at 1.3 ml/min in constant flow mode. The injector temperature was 250°C, the injection volume 1 μl, and the split ratio 1:20. The initial oven temperature of 60°C was held for 1 minute, then increased at a rate of 5°C/min up to 220°C, and subsequently at a rate of 15°C/min up to 280°C, and finally was held isothermal for 1 min. The transfer line to the MSD was set at 280°C and the scan conditions were: M/Z 40–300, at 1.75 scans/sec.

Prior to analysis 900 μl of the volatile fractions were mixed with 100 μl of biphenyl (2.0 mg/ml in hexane) as internal standard. The components of essential oils were identified by comparing their relative retention times and mass spectra with those of Registry of Mass Spectral Data and literature citations [40,41]. The amount of the individual compounds in the fractions was calculated using the Total Ion Current from the MSD signal and assuming the same response as for the internal standard biphenyl.

Inocula containing 10^6^ cells/ml were spread on the medium (Sabouraud with added penicillin (40 units/ml) and streptomycin (2 mg/ml of medium)). The antifungal activity test was carried out by the disk diffusion method using sterilized 6 mm diameter filter paper disks. From each volatile oil preparation a quantity of 50 μl was submitted to testing and the tests were done in triplicate. The inoculated plates were incubated at 35°C for 24 h. Standard bio discs of Nystatin 100 units/disk (Himedia Laboratories Ltd., India) were used as positive control and blank sterilized filter papers as negative control.

After the incubation, the diameter of the inhibition zone for each essential oil was measured in millimeters (including the 6 mm diameter of the disk). The results are expressed as mean values of three determinations ± S.D.

### Antifungal activity *in vitro*

The experimental protocols were approved by the Animal Ethics Committee of the Faculty of Veterinary Medicine Timisoara and conducted accordingly. All 7 *Artemisia* preparations were subjected to the disc diffusion test against *Candida albicans* (ATCC 10231). Inhibition zones observed ranged from 23.5 mm to 13 mm for the essential oils and volatile oils. An 20.5 mm inhibition zone was determined for Nystatin and 6 mm (the diameter of the bio-disc) for the negative control (Table 4).

**Table 4:** **Inhibitory *in vitro *** **effects of the volatile fractions on *Candida albicans*** (**ATCC 10231**)

**Volatile fraction**	**Inhibition zone diameter (mm** **±** **SD)**
*A. dracunculus* (oil)	15.5 ± 1.52
*A. dracunculus* (CH_2_Cl_2_)	13.0 ± 2.08
*A. abrotanum* (oil)	23.5 ± 1.52
*A. abrotanum* (CH_2_Cl_2_)	21.5 ± 3.51
*A. absinthium* (CH_2_Cl_2_)	13.0 ± 1.15
*A. vulgaris* (oil)	16.6 ± 1.57
*A. vulgaris* (CH_2_Cl_2_)	17.0 ± 1.15
Nystatin (positive control)	20.5 ± 4.04
Blank filter paper (negative control)	6.0 ± 0

Highest antifungal activity was observed with *A. abrotanum* (both volatile fractions) followed by *A. vulgaris*, *A. dracunculus*, and *A. absinthium*.

## Conclusions

Since all volatile fractions caused inhibition we conclude that compounds found in different quantities are responsible for the varying *in vitro*-activity against *C. albicans*, but it is difficult to attribute this effect of complex mixtures to a particular constituent. Possibly synergistic as well as antagonistic effects of compounds in the oil should also be taken into consideration as often stronger antifungal activity can be observed with complete essential oils in comparison to single oil components [42]. Our studies showed that different procedures for preparation of volatile fractions, starting from extracts or plant material, resulted in different chemical compositions. This phenomenon may contribute to the contradictory results published for the antimycotic effects of *Artemisia*. Under the conditions of this study, volatile fractions from *Artemisia* plants exert antifungal *in vitro* activity. Our results may open paths for the development of new phytotherapeutic products from the *Artemisia* species studied.

## Abbreviations

C. albicans: *Candida albicans*; GC-MS: Gas chromatography–mass spectrometry.

## Competing interests

The authors declare that they have no competing interests.

## Authors’ contributions

OD, SI and CRT designed the study. OD, SI and CR coordinated the preparation of the manuscript. OD and SK carried out the preparation of the essential oils. CR carried out GC/MS analysis. NI and OD performed the in vitro tests. CV carried out the statistical analyses. All authors contributed to data analysis, read and approved the final manuscript.
